# Mechanisms of ATP release in pain: role of pannexin and connexin channels

**DOI:** 10.1007/s11302-021-09822-6

**Published:** 2021-11-18

**Authors:** Manuel F. Muñoz, Theanne N. Griffith, Jorge E. Contreras

**Affiliations:** Department of Physiology and Membrane Biology, School of Medicine, University of California, Davis, USA

**Keywords:** ATP release, Connexins, Pannexins, Acute pain, Chronic pain

## Abstract

Pain is a physiological response to bodily damage and serves as a warning of potential threat. Pain can also transform from an acute response to noxious stimuli to a chronic condition with notable emotional and psychological components that requires treatment. Indeed, the management of chronic pain is currently an important unmet societal need. Several reports have implicated the release of the neurotransmitter adenosine triphosphate (ATP) and subsequent activation of purinergic receptors in distinct pain etiologies. Purinergic receptors are broadly expressed in peripheral neurons and the spinal cord; thus, purinergic signaling in sensory neurons or in spinal circuits may be critical for pain processing. Nevertheless, an outstanding question remains: what are the mechanisms of ATP release that initiate nociceptive signaling? Connexin and pannexin channels are established conduits of ATP release and have been suggested to play important roles in a variety of pathologies, including several models of pain. As such, these large-pore channels represent a new and exciting putative pharmacological target for pain treatment. Herein, we will review the current evidence for a role of connexin and pannexin channels in ATP release during nociceptive signaling, such as neuropathic and inflammatory pain. Collectively, these studies provide compelling evidence for an important role of connexins and pannexins in pain processing.

## Introduction

Pain is an essential physiological response, warning of current or possible tissue damage, and is also modulated by psychological, emotional, and societal components [1]. Within this definition lies an array of pain etiologies, which can be broadly classified as either acute or chronic. Acute pain, also referred to as nociceptive pain, is defined as a sensation evoked by noxious stimuli that activates pain-sensing peripheral sensory neurons. These peripheral nociceptors transmit acute pain signals via myelinated A-delta- and unmyelinated C-afferents [2, 3]. Nociceptive pain can originate in most tissues, including musculoskeletal, visceral, and skin [4–6]. On the other hand, chronic pain is described as the manifestation of an injury, disorder, or disease that can last for months to years [3]. Two common forms of chronic pain include neuropathic and inflammatory pain. *Neuropathic pain* is defined as pain caused by a lesion or disease of the somatosensory nervous system [7]. A plethora of disorders, or their respective treatment plans, result in peripheral neuropathic pain, including AIDS, diabetes, and cancer [8], and approximately ~ 53% of spinal cord injury patients develop some form of neuropathic pain [9]. In addition to peripheral neuropathy, central neuropathic pain can occur as a result of damage to the central nervous system (CNS), including stroke, encephalitis, and demyelinating disease like multiple sclerosis [10]. *Inflammatory pain*, such as that caused by diseases like arthritis, involves tissue damage that results in the recruitment of immune cells and subsequent release of pro-inflammatory substances, including cytokines and adenosine 5′-triphosphate (ATP). Both neuropathic and inflammatory pain are associated with an elevated sensitivity to innocuous stimuli (such as warm water or changing clothes), as well as hypersensitivity to noxious stimuli, referred to as allodynia and hyperalgesia, respectively [11]. There is abundant evidence that extracellular ATP and other nucleotides have an important role in pain signaling both in the periphery and in the CNS. Nevertheless, important questions remain, including the mechanisms through which ATP is released to activate nociceptive purinergic signaling pathways.

In 1972, ATP was proposed to be an extracellular signaling molecule present in the peripheral nervous system (PNS) and the CNS [12]. Since then, 7 subtypes of ionotropic purinergic receptors have been identified (P2X1-7) [13]. Additionally, 8 subtypes of metabotropic receptors have also been characterized (P2Y1, P2Y2, P2Y4, P2Y6, P2Y11, P2Y12, P2Y13, and P2Y14) [13]. All P2X isoforms have been detected in the PNS [13], whereas only transcripts of P2Y1, P2Y2, P2Y4, and P2Y6 have been observed in sensory neurons of the dorsal root, trigeminal, and nodose ganglia [13–15]. Nearly half of spinal cord dorsal horn neurons use ATP as a fast excitatory neurotransmitter, where it activates P2X receptors present in laminae I–III of the spinal cord, the termination zone of presynaptic nociceptive afferents [16]. Much research has been devoted to establishing the subtype specific contributions of purinergic receptors to different forms of pain signaling. In particular, activation of the homomeric P2X3 and P2X2/3 receptors in sensory neurons have been associated with acute pain behaviors [17–19]. For example, blocking P2X3 receptors with the selective P2X3 receptor antagonist A-317491 prevented acute muscle hyperalgesia, but had no effect on chronic-muscle pain [18]. Conversely, the activation and upregulation of P2X4 receptors on glial cells (i.e., microglia) are linked to the pathogenesis and development of neuropathic pain and mechanical allodynia, thus representing a pathway promoting the development of chronic pain [20–22]. Similarly, P2X7 receptors have been primarily associated with neuropathic and chronic inflammatory pain, showing a selective upregulation in human dorsal root ganglia, glial cells, and immune cells (i.e., monocytes and lymphocytes) [3, 23, 24].

**Fig. 1:**
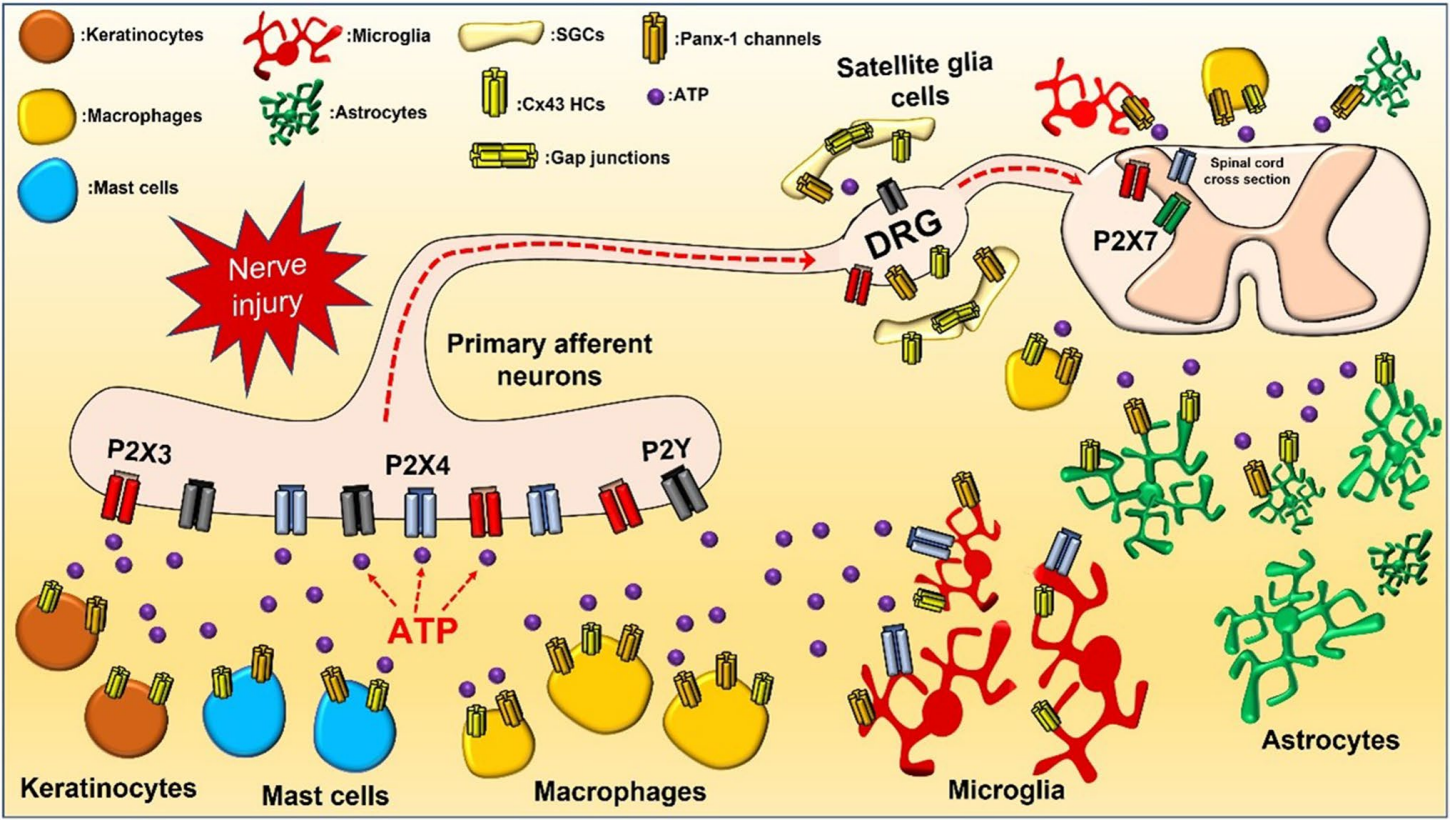
A schematic diagram of the cell types and possible sources of ATP release during pain. ATP is released from a variety of cells, including epithelial cells (keratinocytes), immune cells (mast cells, macrophages), glial cells (microglial, astrocyte, and satellite glial cells), and neurons. Pannexin and connexin channels are shown in the cell types where they have been found to play a role in pain. Similarly, P2X or P2Y receptors are displayed in sensory nerves, DRG, and spinal cord, where they can transmit acute and/or chronic pain signals

Several reviews have recently addressed in detail the roles purinergic receptors play in both acute and chronic pain [25–27]. Conversely, less studied are the mechanisms of ATP release that initiate purinergic signaling during pain. Pannexins and connexin channels have been proposed to be critical for ATP release involved in the development of pain, representing possible therapeutical targets for analgesic drugs [28, 29]. Herein, we will describe the emerging role of these large-pore channels as conduits of ATP release during nociceptive signaling (Fig. 1).

## ATP signaling in pain

ATP was first explicitly hypothesized to initiate nociceptive signaling via activation of purinergic receptors on sensory nerve terminals nearly 30 years ago [30]. Since then, a plethora of studies have used both pharmacological and genetic approaches to delineate the subtype-specific role of purinergic receptors in diverse pain etiologies. Various P2Y metabotropic receptors are expressed in spinal cord astrocytes, microglia, and small nociceptive cells, as well as large-diameter mechanosensory neurons [31]. Increased expression of both P2Y6 and P2Y11 has been observed following spinal nerve ligation, and pharmacologically blocking these receptors in the spinal cord was antiallodynic [32]. Novel antagonists of P2Y14 receptors were reported to ameliorate neuropathic pain in rats subjected to sciatic nerve injury; however, additional studies are required to support this finding [33]. Upregulation of P2Y12 receptor mRNA and protein in activated spinal microglia has been observed in different rodent models of nerve injury and both pharmacological inhibition and genetic deletion of P2Y12 receptors reduced tactile allodynia following nerve injury [34, 35]. Consistent with this, a P2Y12 agonist intrathecally administered to naive animals produced pain behaviors [34], suggesting that P2Y12 signaling in spinal microglia contributes to neuropathic pain. P2Y12 has also been shown to be regulated in satellite glia cells, which encase dorsal root ganglia (DRG) and trigeminal ganglia (TG) neuron soma, following chronic constriction injury (CCI) in rats to mediate both tactile and thermal hyperalgesia [36]. Finally, P2Y2 receptors are also expressed in satellite glia cells of the TG and inhibition of these receptors reduced mechanical allodynia induced by Complete Freund’s adjuvant (CFA), suggesting the potential role of P2Y2 receptors in inflammatory pain [37].

In contrast to the largely non-neuronal role of P2Y receptors during pain signaling, an increasing number of studies have established the role of P2X receptors in the initiation of pain signaling in sensory neurons innervating organs such as skin, tongue, and bladder [38, 39]. P2X3 receptors and P2X2/3 heteromeric receptors are the most prevalent isoforms in sensory neurons, though transcript and protein expression have been reported for all P2Xs, except P2X7 [40–43]. The P2X3 selective antagonist, A-31749, produced a dose-dependent reduction in visceral hypersensitivity in a model of chemically-induced colitis in rats [44], suggesting a contribution of P2X3 receptors to inflammatory pain mediated by vagal sensory afferents. Interestingly, P2X3 expression was elevated in women with endometriosis and endometriotic lesions, compared with control patients, and a positive correlation was reported between the severity of pain and the expression of P2X3 receptors in the endometrium [45]. As noted, P2X7 is not expressed in sensory neurons, but instead is found in spinal cord neurons and motoneurons where it is activated by high concentrations of ATP following nerve injury [46, 47]. Use of the competitive P2X7 receptor antagonist, A-740003, resulted in blockage of IL-1β release from THP-1 cells, a monocyte-like cell line, and attenuation of tactile allodynia in a concentration-dependent manner in two models of neuropathic pain [48]. In a CFA rat model of arthritis, co-administration of the selective P2X7 receptor inhibitor, oxidized ATP (OxATP), significantly decreased chronic inflammatory pain compared to CFA administration alone [49]. Consistent with this notion, daily administration of the non-selective P2X7 antagonist pyridoxalphosphate-6-azophenyl-2[prime],4[prime]-disulfonic acid (PPADS) led to a decrease in observable pain behaviors, reversed mechanical allodynia, reduced expression of proinflammatory cytokines, and decreased neuronal nitric oxide synthase (nNOS) and inducible nitric oxide synthase (iNOS) immunoreactivity in a CCI mouse model [50]. Furthermore, dexmedetomidine, selective α2-adrenoceptor agonist used for sedation, was proposed to attenuate neuropathic pain induced by CCI through inhibition of spinal P2X7 receptor expression [51]. P2X7 receptors have been described to be intimately involved in microglial activation and cancer-induced allodynia, in which the use of siRNA of P2X7 reduced these pain-related effects [52]. Collectively, these studies support a role for P2X7 receptors in the spinal cord as early mediators of cellular damage during chronic inflammatory pain. Nevertheless, important contributions of other P2X isoforms have also been identified.

Indeed, P2X4 may have an exciting new role as a therapeutic target for neuropathic pain in humans with herpes zoster or Guillain-Barré syndrome (GBS). A novel P2X4 antagonist, NP-1815-PX, exhibited anti-allodynic effects in a mouse model of herpetic pain and reduced mechanical allodynia after spinal nerve transection [53]. These analgesic effects may result from decreased mRNA levels of P2X4 receptors in spinal microglial cells, which were observed after inoculation with the herpes virus [53]. Nevertheless, the specificity of this new compound for P2X4 receptors remains to be verified by other groups. In experimental autoimmune neuritis, an animal model of the GBS subtype demyelinating polyradiculoneuropathy, P2X4 receptor expression increased in microglial cells present in the lumbar dorsal horn, but not in astrocytes [54]. Based on immunohistochemistry, the authors suggested that the accumulation of P2X4 receptors in the lumbar dorsal horn may contribute to mechanical allodynia in this model [54]. Consistently, similar results were obtained after GBS animals were treated with the antidepressant paroxetine, which is also a potent antagonist of P2X4, resulting in attenuated mechanical allodynia [55]. Blocking monoclonal antibodies injected intrathecally at the L4-L6 spinal level in mice also induced analgesia in a mouse model of sciatic nerve ligation [56]. In line with this, global P2X4^−/−^ mice did not develop mechanical hypersensitivity after peripheral nerve injury, and also had impaired brain-derived neurotrophic factor (BDNF) release [57]. A different study, however, showed chronic inflammatory and neuropathic pain signaling were intact in P2X4^−/−^ mice, which suggests a pain-etiology specific role for this isoform [58]. Collectively, these results highlight the important role of P2X receptors in various pain signaling pathways and demonstrate that purinergic signaling represents an exciting therapeutic target for pain treatment. Conversely, the mechanisms mediating ATP release during pain remain poorly defined.

## Sources of ATP release in pain

Several cell types are poised to release ATP to orchestrate the complex signaling pathways that underlie nociception (Fig. 1). Sensory neurons play a fundamental role in nociception by transmitting pain signals from the periphery to the CNS. Primary afferents also release ATP onto lamina II spinal cord neurons to activate post-synaptic P2X receptors and elicit fast excitatory postsynaptic currents (EPSCs), which can be blocked by antagonists, such as PPADS and suramin [59]. Work with mice harboring genetic deletion of the ATP vesicular transport protein, vesicular nucleotide transporter (VNUT), suggests a role for vesicle-mediated ATP release during different forms of pain. VNUT, whose gene name is *SLC17A9*, was originally cloned from mouse and human and was postulated to play a critical role in ATP transport in ATP-releasing cells [60]. It was shown that siRNA-mediated knockdown of *SLC17A9* in PC12 cells, a pheochromocytoma cell line, diminished ATP exocytosis, suggesting its participation in ATP release from secretory vesicles [60]. VNUTs have been functionally linked to ATP exocytosis in a variety of physiological processes, including lysosomal ATP accumulation, cell survival, neutrophil migration, astrocyte signaling, neuro-glial communication, and microglial-mediated neuropathic pain [61–69]. Interestingly, the use of clodronate, a selective VNUT inhibitor, attenuated chronic inflammatory and neuropathic tactile pain in wildtype mice, but not in VNUT^−/−^ animals, with VNUT^−/−^ mice also showing reduced mechanical hyperalgesia at baseline compared to controls [65]. Clodronate treatment decreased ATP release, and reduced expression of inflammatory markers, IL-6 release, and edema, supporting the role of VNUT mediating chronic neuropathic pain [65]. Consistent with this, spinal dorsal horn neurons from VNUT^−/−^ mice did not show increased ATP release following nerve injury, which was observed in control animals, providing evidence that pain-induced exocytotic ATP release in the spinal cord is dependent on VNUT [63]. There was no difference, however, between VNUT^−/−^ mice and controls in acute nocifensive behaviors, suggesting this pathway is specifically activated during chronic pain conditions. Furthermore, tactile allodynia was still present when VNUT was selectively deleted in astrocytes, microglia, and primary sensory neurons after peripheral nerve injury [63]. This suggests that vesicular ATP release does not trigger pain signaling in all cell types involved in pain, opening the possibility for non-vesicular ATP release mechanisms, such as those mediated by molecule permeable channels like connexin-43 (Cx43) and pannexin-1 (Panx-1) hemichannels.

Epidermal keratinocytes have an intimate physical interaction with intraepidermal nociceptive nerve fibers and are thus poised to modulate pain signaling [70, 71]. These cells express several transient receptor potential (TRP) channels that respond to a variety of environmental signals, including noxious stimuli [72–74]. Cultured human epidermal keratinocytes stimulated with capsaicin, the spicy component of chili peppers, responded with increased Ca^2+^ signaling [75]. Topical application of capsaicin to the mouse hind paw induced ATP release from keratinocytes, along with the expression of the neuronal activation marker, c-fos, in laminae I and II of the dorsal horn [76]. Keratinocytes are capable of non-vesicular ATP release via connexin hemichannels following air stimulation, a model of mechanical force, thus demonstrating the potential role for these cells to initiate purinergic signaling in primary afferents. ATP release from keratinocytes has also been shown to induce Ca^*2*+^ waves in dorsal root ganglion (DRG) neurons, leading to pain behaviors [77–80]. Importantly, it has been shown that mechanical stimulation of primary normal human epidermal keratinocytes (NHEKs) causes an increase in Ca^2+^ signaling in DRG neurons, which was diminished in the presence of ATP-degrading enzyme apyrase, as well as P2X receptor blockers suramin and PPADS, suggesting that ATP release from keratinocytes onto DRG neurons may represent an important pathway of nociceptive transduction during pain [78].

Mast cells are granulated hematopoietic cells that are part of the immune and neuroimmune systems and play a crucial role as first responders during several painful pathologies [81, 82]. When these cells are activated and consequently degranulated, they secrete several pro-inflammatory molecules locally at the site of the injury, such as ATP, TNF-α, and interleukins that can amplify nociceptor activation and pain signaling. This occurs via direct activation of sensory axons, as well as microglia and other mast cells [82–84]. Macrophages are another important cell class involved in pain signaling that release ATP, as well as cytokines and TNF-α. These pro-inflammatory signals are detected by nociceptors and contribute to peripheral neuropathic pain pathogenesis [85–88]. Indeed, administration of macrophage depleting agents suppresses thermal hyperalgesia and tactile allodynia induced by the partial SNL in male mice [89].

Finally, glial cells are one of the most important non-neuronal cell types that release ATP and are involved in pain signaling pathways. Astrocytes are thought to be critical targets for the late maintenance phase of neuropathic pain after nerve injury or tissue damage [90], due to their activation by glutamate, ATP, calcitonin gene-related peptide (CGRP), or substance P [91], which results in astrocytic release of pro-inflammatory cytokines [92]. Nerve injury produces astrogliosis and ATP release from astrocytes in the injured area, which subsequently leads to neuroinflammation [93–95]. Furthermore, optogenetic activation of spinal astrocytes evokes mechanical allodynia, thermal hyperalgesia, and pain hypersensitivity via the release of ATP [96]. As with astrocytes in the CNS, satellite glial cells (SGCs) surround the soma of DRG neurons where they are proposed to help control the local cellular environment. SGCs are also important for neuronal health and serve a protective function, much like brain astrocytes. Bidirectional SGC-to nociceptor signaling via ATP release may modulate afferent firing [97, 98], suggesting a role in nociception. Lastly, microglial cells are defined as macrophages of the CNS that undergo an activation process of proliferation [99]. In response to nerve injury in the PNS, infiltrating monocytes differentiate into microglia-like cells [97], upregulating P2X4 receptors [100]. Interestingly, pharmacological blockage of spinal P2X4 receptors reversed tactile allodynia induced by nerve injury, which increased P2X4 expression only in microglial cells, but not in neurons or astrocytic cells [20]. Additionally, microglia have also been proposed to induce ATP release via connexin hemichannels and pannexin channels in the context of physiological neuron-astrocyte-microglial crosstalk [101]. This remains to be examined, however, in the context of pain.

Following ATP release, its availability is tightly regulated by ectonucleotidases, ectoenzymes that regulate extracellular ATP concentrations and, therefore, are closely involved in the temporal kinetics and amplitude of purinergic receptor activation [102]. Ectonucleotidases breakdown ATP into adenosine-5′-diphosphate (ADP), adenosine monophosphate (AMP), and adenosine [103]. They include the nucleoside triphosphate diphosphohydrolase (NTPDase), nucleotide pyrophosphatase/phosphodiesterase (NPP), ecto-50-nucleotidase/CD73, tissue-nonspecific alkaline phosphatase (TNAP), and prostatic acid phosphatase (PAP) families, as well as other phosphatases such as adenosine deaminase (ADA) and purine nucleoside phosphorylase (PNP) [102]. Recently, ectonucleotidases have also been linked to nociceptive signaling. For example, it was reported that sciatic nerve transection produced decreased mRNA levels of the ectonucleotidase PAP in rat DRG, which is consistent with the antiallodynic effect of intrathecal injection of PAP protein in a spared nerve injury [104]. Similarly, a study using a mouse model of resiniferatoxin (RTX)-induced neuropathic pain showed that exogenous PAP treatment via intraperitoneal injection attenuated mechanical allodynia in a dose-dependent manner [105]. This is in line with several other studies demonstrating the anti-nociceptive effects of PAP and the ectonucleotidase CD73 in models of chronic inflammatory or neuropathic pain [106–108]. Thus, mounting evidence suggests ectonucleotidase-mediated control of ATP availability could play an important role in both acute and chronic purinergic nociceptive signaling.

## Biology of connexins and pannexins and its role in ATP release

In humans, there are 21 different connexin (Cxs) isoforms that are found throughout the body. These proteins form hemichannels or gap junction channels that mediate intercellular molecular communication in a diverse array of processes. These include various aspects of development and physiology, as well as responses to injury and inflammation [109]. In a cell, six connexin proteins oligomerize to form a hemichannel that is sorted to the plasma membrane. The docking of two hemichannels at the plasma membrane from adjacent cells leads to the formation of gap junction channels (GJCs). GJCs constitute intercellular channels that allow the cytoplasmic passage of second messengers such as Ca^2+^ and inositol 1,4,5-trisphosphate (IP_3_) between neighboring cells [110–112]. Undocked hemichannels can also open at the plasma membrane and play paracrine or autocrine roles by mediating communication between the extracellular and intracellular space via the release of transmitter molecules such as NAD^+^, glutamate, prostaglandins, and ATP [113–116].

An interesting demonstration for ATP permeability via Cx43-formed hemichannels combined single-channel recordings along with the luciferace/luciferine bioluminescence assay of ATP release [117]. The authors showed that ATP release occurs in parallel with hemichannel opening in the C6 rat glioma cell line. ATP release was not observed when hemichannels were closed or in the presence of hemichannel blockers such as carbenoxolone (CBX) and 5-nitro-2-(3-phenylpropylamino) benzoic acid (NPPB) [117]. ATP release via connexin hemichannels has been proposed to have both physiological and pathological roles. It has been shown that after a cerebral ischemia and reperfusion model of oxygen and glucose deprivation, primary astrocyte cultures have increased extracellular ATP levels and ethidium uptake, which was prevented in the presence of Cx43 blocking peptides, Gap19 and Gap26, suggesting that Cx43 hemichannel mediates the release of ATP after brain ischemia [118]. Interestingly, photostimulation of Hensen’s inner ear cell cultures with caged-IP_3_ induces the release of ATP, an effect that was not observed in Cx26^−/−^ or Cx30^−/−^ cell cultures, providing evidence that ATP is also released from connexin-hemichannels in sensory hair cell regions [119]. Consistent with this notion, Cx26 and Cx30 missense mutations that cause hearing-impairments, deafness, and skin disorders significantly alter hemichannel-mediated ATP release [120–122]. Interestingly, Cx43, Cx30, and Cx26 are expressed in astrocytes, with Cx43 being the most predominant subtype [123, 124]. Cultured microglia cells displayed increased expression levels of Cx29, Cx32, Cx36, and Cx46 in proinflammatory conditions induced by lipopolysaccharides (LPS), TNF-α, or brain injury [125]. Mast cells have been shown to express Cx43 protein, while macrophages were found to express both Cx43 and Cx37 [126]. Activated SGCs surrounding trigeminal ganglia neurons also have high levels of Cx43 expression after lower first molar pulp inflammation, which was inhibited by the blocking peptide of Cx43, Gap26 [127]. RNA sequencing analysis showed that Cx43, Cx32, Cx30, Cx26, Cx45, and Cx36 are predominantly expressed in the trigeminal ganglia and DRG of adult mice [128, 129]. Thus, there is a growing body of evidence indicating that cells involved in pain signaling and modulation express various types of connexin proteins, which can potentially serve as an important purinergic pathway for nociceptive signaling.

In 2000, Sergey Lukyanov’s group discovered pannexin proteins, which were highly similar to invertebrate innexins [130]. In the human genome, 3 genes encode for pannexins: Panx-1, Panx-2, and Panx-3 [131]. Pannexins are considered integral membrane proteins, with cytoplasmic amino- and carboxy-terminals, four transmembrane segments, and two extracellular loops. This topology is grossly similar to that observed for connexin proteins [132]; however, pannexin channels do not form GJCs, likely due to extracellular glycosylation. They do form plasma membrane channels that allow for the passage of substances up to 1 kDa in size [133–135]. Recent high-resolution structures show that the Panx-1 channel is formed by oligomerization of seven Panx-1 proteins [136–138]. Pannexins were extensively detected in the brain, gastrointestinal tract, spinal cord, lung, kidney, thyroid, skeletal muscle, heart, and endocrine organs [133]. Panx-1 is expressed in a variety of cell types, including lymphocytes, astrocytes, neurons, microglia, adipocytes, airway epithelia, and blood vessels [139–143]. Conversely, Panx-2 is preferentially expressed in the CNS and Panx-3 is expressed in skeletal muscle and the epidermal layer of skin [144, 145]. The most well-studied pannexin is Panx-1, which has been shown to play essential roles in several physiological processes such as maturation of excitatory synapses [146], bone differentiation [147], and skin development [148]. Panx-1 channels are also linked to various pathophysiological roles including neuronal death [149], hemodynamic response to hypoxia [150], and neuroinflammation [151]. While many reports have established that Panx-1 channels are permeable to ATP, it has been suggested that only certain open channel conformations will permit the permeation of large molecules [152]. Currently, available high-resolution structures of open Panx-1 channels are compatible with chloride permeability, but it is unlikely that their pore, with the dimensions reported, can permit passage of large molecules like ATP. Nevertheless, it is likely that Panx-1 channels adopt various open conformations, as they display multiple channel conductances that range from 50 to 500 pS [152–155]. It is probable that only the larger unitary conductance (~ 500 pS) can permeate ATP and other metabolites, whereas smaller channel conductances are primarily permeating chloride ions, as has been suggested [135, 152]. However, Panx-1 channels in which the C-terminus is deleted by caspases displayed ATP permeability with channel conductances ranging from 50 to 100 pS [155]. This has been recently corroborated by studies using purified Panx-1 channels in proteoliposomes, which upon activation with caspase-3 became permeable to large molecules (up to 1 kDa) including ATP and glutamate [156]. Importantly, most of the cells described previously as pain mediators such keratinocytes, mast cells, macrophages, astrocytes, microglia, and SGCs have been reported to release ATP via pannexin-based channels [157–162]. Collectively, this evidence indicates that connexin and pannexin proteins may represent a putative therapeutical target to dampen ATP release from cells, which could be important for attenuating the generation and development of different forms of pain.

## Connexin 43 hemichannels and gap junctions in nerve injury-induced pain

Among the different connexin subtypes, Cx43 is by far the most well studied in various pain models. It is ubiquitously expressed, and therefore is found in many cellular types that are involved in pain signaling. This includes glial cells, such as astrocytes of the spinal cord dorsal horn [163], and SGCs in the trigeminal ganglion [164]. Several reports have suggested a particular role for ATP release from Cx43 hemichannels in pain evoked by nerve injury. Mechanical allodynia and heat hyperalgesia were significantly reduced in knockout Cx30/Cx43 mice compared to controls during a two-month period in a weight drop spinal cord injury model. Astrogliosis was also reduced in these genetically modified mice after 1 week and 1-month post-injury when compared to wild-type controls. Interestingly, deletion of Cx30 alone does not prevent mechanical allodynia and hyperalgesia [163]. Using immunofluorescence analysis, a subsequent study found that following nerve injury Cx43 co-localized with the reactive astrocytic marker, glial fibrillary acidic protein (GFAP), but not with the neuronal marker NeuN, or the microglial/macrophage marker CX3C chemokine receptor 1 (CX3CR1) [165]. Cx43 protein expression was increased in the spinal cord dorsal horn at 10 and 21 days following nerve injury and, interestingly, intrathecal injection of Cx43 blocking peptides, ^43^Gap26 or ^37,43^Gap27 significantly reduced mechanical allodynia in this model [165]. The authors also showed that Cx43 hemichannels promote the release of the proinflammatory chemokine CXCL1 in response to spinal injection of TNF-α. This consequently enhanced excitatory synaptic transmission in spinal cord neurons, thereby promoting mechanical allodynia for > 48 h. This is further evidence that Cx43-based hemichannels could represent an important pathway for pain signaling.

Adding further complexity to the participation of connexin proteins in pain, pharmacological studies have suggested not only the involvement of the connexin-based hemichannels in the development of pain, but also the role of intercellular gap junction channels. It was reported that intrathecal administration of CBX, also used as a non-specific gap junction decoupler, reverted the generation of mechanical allodynia and thermal hyperalgesia in a sciatic inflammatory neuropathy model, as well as in the classic model of CCI in adult male rats [166]. Similarly, L4 DRG neurons treated with CFA or animals with CCI presented a higher number of coupled neurons (defined as two or more neuronal soma located within 1 mm of each other showing synchronous Ca^2+^ GCaMP-coded signals) compared with the vehicle using in vivo DRG Ca^2+^ imaging [167]. Notably, Cx43 expression is increased after CFA and CCI only in SGCs of the DRG. Consistently, the specific deletion of Cx43 in SGCs showed an attenuation of Ca^2+^ coupled cells and less mechanical hyperalgesia induced by CFA in comparison to control animals, suggesting a critical role of Cx43 SGCs in pain. Systemic administration of CBX also induced a significant decrease in coupled neurons and pain hypersensitivity, similarly with results performed in Cx43 KO mice. This suggests that Cx43 in SGCs contributes to neuronal coupling via intercellular communication and are fundamental for the development of mechanical hyperalgesia and allodynia [167]. Interestingly, it has been reported that following injection of the dye Lucifer Yellow in mice with sciatic nerve neuritis, SGCs showed an increased number of coupled cells in relation to control conditions. Furthermore, based on electron microscopy data, SGCs from animals with nerve injury presented ultrastructural changes compared to wild-type animals, expressing an increased number of gap junction plaques and bridges between sheaths surrounding neurons, supporting the finding of an enhanced gap junction coupling signaling after nerve injury [168]. In other models of inflammatory pain, intraperitoneal administration of CBX reduced tactile hypersensitivity induced by injection of CFA in the submandibular skin of mice [169]. Increasing doses of CBX inhibited evident symptoms of neuropathic pain, such as mechanical and heat hypersensitivity. Furthermore, 30–60 min after topical application of CBX to the spinal cord, the number of action potentials evoked during extracellular recordings from dorsal horn neurons was reduced [170]. Although Cx43 gap junction channels do not directly leak ATP to the extracellular environments, they seem to be important modulators of chronic pain likely via the passage of the ions and signaling molecules [171, 172]. Indeed, intercellular Ca^2+^ waves that are spread via astrocytic Cx43 gap junction channels [173] can contribute to the release of ATP from astrocytes onto sensory neurons, which consequently may trigger the activation of purinergic receptors on nociceptor terminals [163]. Overall, the findings suggest that Cx43-mediated cell–cell coupling might have a significant effect on the hyperexcitability of sensory neurons in chronic pain. Yet, CBX and genetic deletion of Cx43 also affect activity of Cx43 hemichannels; thus, a role for hemichannels cannot be ruled out in these models.

Connexin proteins have also been linked to chemotherapy-induced neuropathic pain [174, 175]. In fact, animals treated with bortezomib, a chemotherapy drug that induces peripheral neuropathy, exhibit increased expression of Cx43 in spinal astrocytes compared to those treated with vehicle [175]. Co-treatment with CBX reduced alterations in mechanical sensitivity, which lead to the hypothesis that upregulation of Cx43 by treatment with bortezomib might pathologically enhance hemichannel and GJC activity [176]. This finding is also replicated with oxaliplatin treatment, another chemotherapy drug that often produces peripheral neuropathy. Oxaliplatin treatment increased protein expression of GFAP and Cx43 in spinal cord astrocytes at day 7 post-administration, whereas Cx32 and Cx36 protein expression were unmodified compared to vehicle-treated animals [175]. Application of CBX produced a decrease in mechanical hypersensitivity triggered by oxaliplatin [175]. Consistent with the pathological role described above for augmented cell-to-cell coupling in SGCs, in vitro studies showed that oxaliplatin induced an increased incidence of cell coupling in cultured SGCs compared with those in control conditions [177]. While it is clear that chemotherapy drugs affect Cx43 expression, the specific role that gap junctions and hemichannels play in neuropathic pain induced by chemotherapy treatment needs to be explored further using more specific pharmacological and genetic approaches.

## Critical role of Panx-1-based channels in neuropathic pain

Pannexin channels, particularly those formed by Panx-1 proteins, have been implicated in neuropathic pain and several other pathophysiological conditions [161]. Low doses of CBX (10, 25, and 50 mg/kg), generally used as a Panx-1 channel blocker [178], were reported to efficiently reverse the hypersensitivity induced by CFA [169], compared with saline-treated animals. Consistent with this, Panx-1 null mice did not develop tactile hypersensitivity in an orofacial pain model compared with wild-type animals. Spray and colleagues showed that submandibular injection of CFA induced an increase in mRNA levels of Panx-1, IL-1, caspase-1, and increased ATP release in the trigeminal ganglia. All of these effects were completely abolished in animals with a global knockout of Panx-1. This was replicated in mice with Panx-1 deletion only in GFAP-positive glia cells, highlighting the importance of Panx-1 channels in the regulation of pain responses [179].

Interestingly, it has been reported that pain hypersensitivity evoked by peripheral nerve injury depends on diurnal oscillations of glucocorticoid release from the adrenal glands [180]. In this study, the authors established that temporal elevations in glucocorticoid levels increase the release of ATP in the spinal cord, which could have a direct effect on the activation of P2Y12 purinergic receptors present on the cell membrane of microglial cells. Four-hour treatment with corticosterone induced an increase in ATP release from primary cultures of astrocytes compared to vehicle-treated astrocytes, which was reduced by pannexin channel inhibitors, but not connexin channel inhibitors [180]. For instance, astrocytes incubated with 100 µM ^10^Panx-1 or astrocytes transfected with a siRNA against pannexin-1 has significantly decreased ATP release induced by corticosterone. Therefore, the authors suggest that corticosterone-induced ATP release occurs through a mechanism that depends on Panx-1 channel opening, leading to the activation of P2Y12 receptors on microglia to generate mechanical allodynia [180].

In other models of neuropathic pain, such as L5/L6 SNL, similar results have been obtained. For example, nerve injury produces an increase in Panx-1 expression in DRG at 5, 10, and 21 days after the SNL surgery. Similarly, intrathecal injection of CBX and ^10^Panx-1 reduced pain hypersensitivity, in line with results using siRNA knockdown of Panx-1, in which tactile and pressure withdrawal thresholds were decreased in SNL-treated rats 3 weeks post-surgery [181]. Similar results have shown that intrathecal administration of the Panx-1 blockers ^10^Panx-1, carbenoxolone, and probenecid depressed the spinal C-reflex wind-up activity and mechanical hyperalgesia in neuropathic rats 10 days after nerve injury [182]. Again, this supports the notion that Panx-1 channels are mediators of ATP release in neuropathic pain syndromes. In other pathologies, such as cortical spreading depression (CSD), which is purported to be the cause of migraine aura and headaches, inhibition of Panx-1 channels with CBX suppressed trigeminal pain fiber activation, degranulation of mast cells, and pain as measured using a mouse grimace scale [183]. Additionally, Panx-1 channels have been suggested to be targets of opiates. The withdrawal behavior evoked by morphine normally induces long-term facilitation in neurons located at lamina I and II of the spinal dorsal horn, generating analgesia. However, genetic deletion of Panx-1 in microglia abolished the spinal facilitation and ameliorated the withdrawal response. The investigators suggested that during withdrawal, Panx-1 channels are activated, which induces ATP release from microglial cells [160]. Taken together, this large body of evidence indicates that Panx-1 channels represent a critical pathway to induce the release of ATP from different inflammatory cells that contribute to the pain signaling.

## Future directions

During pain, a plethora of cells, epithelial cells, glia, and immune cells mediate the release of ATP to activate purinergic receptors on sensory terminals and the subsequent transmission of nociceptive signals to the spinal cord and brain. Evidence suggests that during different pain states, connexin and pannexin channels might serve as critical conduits for ATP release, as well as other pro-inflammatory molecules. Nevertheless, the role for these channels in most pain animal models is mainly supported by pharmacological approaches. These include selective and non-selective blockers such as the mimetic blocking peptide ^10^Panx-1, probenecid, and, in numerous cases, CBX. While the selectivity of mimetic blocking peptides is to some extent reliable, non-selective blockers such as CBX in the absence of other indistinct treatments make results more complex to interpret. For instance, there are other ATP-permeable channels such as the calcium homeostasis modulators (CALHMs) or volume-regulated anion channels (named SWELL1 or LRRC8A), which share pharmacological properties with pannexin and connexin channels. Importantly, a putative role for these channels in pain also remains unexplored. Thus, future studies are needed using more specific pharmacological tools and cell type–specific knockout rodent models for connexin and pannexin genes. These studies will help us to unequivocally determine the potential therapeutical role of pannexins and connexin proteins for treating pain, which is a broad and complicated condition among patients around the world, representing a difficult yet important public health challenge.

## Data Availability

The datasets analyzed during the current study are available from the corresponding author on reasonable request.

## Code availability

No applicable.
